# Leptin Matures Aspects of Lung Structure and Function in the Ovine Fetus

**DOI:** 10.1210/en.2015-1729

**Published:** 2015-10-19

**Authors:** Miles J. De Blasio, Maria Boije, Sarah L. Kempster, Gordon C. S. Smith, D. Stephen Charnock-Jones, Alice Denyer, Alexandra Hughes, F. B. Peter Wooding, Dominique Blache, Abigail L. Fowden, Alison J. Forhead

**Affiliations:** Department of Physiology, Development and Neuroscience (M.J.D.B., M.B., A.D., A.H., F.B.P.W., A.L.F., A.J.F.), University of Cambridge, Cambridge CB2 3EG, United Kingdom; Department of Medicine (S.L.K.), University of Cambridge, Addenbrooke's Hospital, Cambridge CB2 0QQ, United Kingdom; Department of Obstetrics and Gynaecology (G.C.S.S., D.S.C.-J.), University of Cambridge, The Rosie Hospital, Cambridge CB2 0SW, United Kingdom; School of Animal Biology (D.B.), University of Western Australia, Crawley, Perth, Western Australia, Australia 60095; and Department of Biological and Medical Sciences (A.J.F.), Oxford Brookes University, Oxford OX3 0BP, United Kingdom

## Abstract

In human and ovine fetuses, glucocorticoids stimulate leptin secretion, although the extent to which leptin mediates the maturational effects of glucocorticoids on pulmonary development is unclear. This study investigated the effects of leptin administration on indices of lung structure and function before birth. Chronically catheterized singleton sheep fetuses were infused iv for 5 days with either saline or recombinant ovine leptin (0.5 mg/kg · d leptin (LEP), 0.5 LEP or 1.0 mg/kg · d, 1.0 LEP) from 125 days of gestation (term ∼145 d). Over the infusion, leptin administration increased plasma leptin, but not cortisol, concentrations. On the fifth day of infusion, 0.5 LEP reduced alveolar wall thickness and increased the volume at closing pressure of the pressure-volume deflation curve, interalveolar septal elastin content, secondary septal crest density, and the mRNA abundance of the leptin receptor (Ob-R) and surfactant protein (SP) B. Neither treatment influenced static lung compliance, maximal lung volume at 40 cmH_2_O, lung compartment volumes, alveolar surface area, pulmonary glycogen, protein content of the long form signaling Ob-Rb or phosphorylated signal transducers and activators of transcription-3, or mRNA levels of SP-A, C, or D, elastin, vascular endothelial growth factor-A, the vascular endothelial growth factor receptor 2, angiotensin-converting enzyme, peroxisome proliferator-activated receptor γ, or parathyroid hormone-related peptide. Leptin administration in the ovine fetus during late gestation promotes aspects of lung maturation, including up-regulation of SP-B.

Maturation of the fetal lungs close to term is essential for normal respiratory function at birth and the successful transition from a placental to a pulmonary supply of oxygen. A number of structural and functional changes occur in the fetal lungs, including development of the diffusion exchange surface by thinning of alveolar septa, vascular remodeling and removal of lung liquid; expression of elastin and activation of surfactant synthesis and secretion in type II pneumocytes to improve lung compliance; and up-regulation of angiotensin-converting enzyme (ACE) activity to induce bioavailability of angiotensin II (1–4). Preterm delivery before these changes take place often leads to respiratory distress syndrome (RDS) in the neonate. In England and Wales, prematurity affects about 7% of all births and around 24 babies die each week as a result of respiratory and other complications arising from preterm birth (5).

Glucocorticoids are well known to promote maturation of the fetal lungs (3). Close to term, a rise in cortisol in the fetal circulation stimulates structural and functional maturation of the lungs in preparation for birth. In addition, antenatal synthetic glucocorticoid treatment, such as dexamethasone, administered to pregnant women at risk of preterm delivery, has markedly reduced neonatal mortality and the incidence of RDS in premature infants (6, 7). The maturational actions of endogenous and exogenous glucocorticoids on pulmonary structure and function in utero may be direct and/or indirect. Glucocorticoid response elements have been identified in several of the genes essential for the production of surfactant (3). In addition, the effects of glucocorticoids on pulmonary maturation may be mediated by other endocrine and/or paracrine systems. For example, the rise in pulmonary ACE concentration induced by glucocorticoids in fetal sheep near term depends on an intact thyroid gland and up-regulation of thyroid hormone activity (8).

Previous studies have suggested that leptin may act as a hormonal signal to mediate, at least in part, the maturational effects of glucocorticoids in utero. In fetal sheep, adipose leptin mRNA abundance and plasma leptin concentration increase towards term in association with the prepartum cortisol surge (9, 10). Developmental increments in leptin synthesis and secretion are abolished by removal of the fetal adrenal gland and prevention of the prepartum cortisol surge, and can be induced prematurely by endogenous and exogenous glucocorticoid treatment (9, 10). Clinical studies have also shown a significant positive relationship between umbilical concentrations (in the fetal circulation) of leptin and cortisol in newborn full-term human infants, independent of gender, birth weight and gestational age (11), and that umbilical leptin concentrations are increased 3-fold in preterm infants delivered after their mothers were treated with antenatal glucocorticoids (12).

Leptin receptors (Ob-R), including the long-form signaling isoform Ob-Rb, are expressed in the fetal lungs of a variety of mammalian species (13–16), and studies in vitro using fetal tissue suggest that leptin may be an important regulator of pulmonary development before birth. Leptin treatment increases surfactant protein (SP) mRNA and protein levels in lung explants from fetal rats (17), and promotes surfactant phospholipid synthesis in type II pneumocytes isolated from fetal rats and rabbits (18, 19). However, the role of leptin in the control of lung development in vivo is unclear, particularly in species like the sheep and human where alveolar formation begins before birth. Therefore, this study examined the effects of an iv infusion of recombinant ovine leptin on structural and molecular indices of pulmonary development in the chronically catheterized fetal sheep. It was hypothesized that leptin exposure in utero would increase static lung compliance, promote thinning and expansion of the alveolar diffusion surface and up-regulate gene transcript levels of elastin, SPs, and ACE. The study also examined the effects of leptin on local factors known to influence lung development, and specifically type II pneumocyte function, such as vascular endothelial growth factor (VEGF)-A and its receptor (VEGFR2), peroxisome proliferator-activated receptor (PPAR)γ, and parathyroid hormone-related peptide (PTHrP) (20–22).

## Materials and Methods

### Animals

Thirty pregnant Welsh Mountain ewes of known gestational age and carrying singleton fetuses were housed in individual pens and fed concentrates (200 g/d; 18% protein and 10 MJ/kg; Sheep Nuts 6; H&G Beart) with hay, water, and a mineral block ad libitum. The animals consumed between 8–11 MJ/d of metabolizable energy and were weighed on the day of surgery. All surgical and experimental procedures were carried out in accordance with the United Kingdom Animals (Scientific Procedures) Act 1986 and were approved by the local animal ethics committee at the University of Cambridge, United Kingdom.

### Surgical procedures

The pregnant ewes were fasted for 18–24 hours before surgery but had free access to water. At between 118 and 120 days of pregnancy (term ∼145 d), the ewes were anesthetized with halothane (1.5–2.0% in O_2_-N_2_O) and positive pressure ventilation. Intravascular catheters were inserted into the femoral artery and vein of the fetus and the femoral artery of the ewe using techniques described previously (23). The catheters were exteriorized and secured in a bag sutured to the flank of the ewe. At surgery, all fetuses were administered with 100-mg ampicillin iv (Penbritin, Beecham Animal Health) and 2-mg gentamycin iv (Frangen-100; Biovet). The ewes were administered with antibiotics im (procaine penicillin, Depocillin, Mycofarm) on the day of surgery and for 3 days thereafter.

### Experimental procedures

#### Fetal leptin infusion and blood sampling

Starting at 124–125 days of gestation, the fetuses were divided randomly into 3 treatment groups and infused iv for 5 days with either saline (0.9% saline, n = 13, 6 male and 7 female) or recombinant ovine leptin at 2 doses, 0.5 mg/kg · d (0.5 LEP; n = 10, 5 male and 5 female) and 1.0 mg/kg · d (1.0 LEP; n = 7, 3 male and 4 female; Protein Laboratories) (24). The infusions were administered via the fetal venous catheter using a Graseby portable infusion pump held in the catheter bag and set at a rate of 3 mL/d. Arterial blood from the fetus and ewe (3 mL) was collected daily from 2 days before and throughout the 5 days of infusion.

Arterial blood gas status was assessed in the fetal and maternal blood samples using an ABL330 Radiometer analyzer (Radiometer) corrected for fetal body temperature to measure pH, partial pressure of oxygen and carbon dioxide, and using an OSM2 hemoximeter (Radiometer) for hemoglobin content and O_2_ saturation. Arterial blood glucose and lactate concentrations were measured using a 2300 Statplus autoanalyser (YSI). All blood samples collected were placed into EDTA-containing tubes and centrifuged at 1000*g* and 4°C for 5 minutes. The plasma samples were stored in aliquots at −20°C until analysis.

### Postmortem procedures

On the fifth day of infusion (129–130 d of gestation), the fetuses were delivered by Caesarean section with the ewe under general anesthesia (20-mg/kg sodium pentobarbitone iv). At delivery, umbilical arterial blood (10 mL) was collected from the fetus before the administration of a lethal dose of barbiturate to both the ewe and fetus (200-mg/kg sodium pentbarbitone iv). The fetus was weighed and a lung function test was performed immediately. In all fetuses, body weight and crown-rump-length (CRL) were measured and used to calculate body mass index (BMI) (kg/m^2^) and ponderal index (PI) (kg/m^3^). The placental cotyledons were dissected, weighed, and categorized into placentome types A, B, C, and D, based on the degree of eversion of the hemophagous zone: the hemophagous zone (the area of extravasated maternal blood at the maternal-fetal interface) is inverted into the type A placentomes and everted over the tissue of the type D placentome with intermediate morphology in types B and C (25). The relative proportions of the placentome types are known to be influenced by circulating glucocorticoids in the sheep fetus (25). Classification of placentome type was carried out by a single investigator (F.B.P.W.). The remaining placental membranes and uterus were also weighed.

### Lung function test

Immediately after the fetus was delivered, lung liquid was drained by gravity, a tracheotomy was performed to intubate the trachea, and the lungs were exposed via a ventral thoracotomy. Static lung compliance was measured with the fetus in the supine position using a water manometer. The lungs were inflated to a maximal luminal pressure of 40 cmH_2_O and then progressively deflated in 5 cmH_2_O steps while recording the volume on a syringe, until return to zero pressure. Maximum volume at 40 cmH_2_O (V_40_) and volume at 0 cmH_2_O (volume at closing pressure) were measured from the pressure-volume curves for each fetus. Static lung compliance was calculated as the slope of the deflation limb of the pressure-volume curve between 10 and 30 cmH_2_O. After static compliance was measured, the fetal lungs were weighed and dissected. The right upper lobe was isolated and inflation-fixed with 4% paraformaldehyde (with 0.2% gluteraldehyde in 0.1M phosphate buffer; pH 7.4) at a pressure of 20 cmH_2_O for at least 30 minutes. The inflated lobe was ligated to maintain the perfusion-fixed pressure, and the volume was determined by water displacement. The lobe was then placed in a container of fresh fixative for 2 weeks. The left upper lobe of the fetal lung was weighed, freeze dried overnight and reweighed to determine pulmonary water content. Samples from the lower right lobe were frozen in liquid nitrogen and stored at −80°C for subsequent molecular analyses and Western blotting.

### Stereological analyses

After 2 weeks in fixative, a 1.0 × 1.0 × 0.5-cm cuboid was cut from the inflated lung lobe; the volume was determined by water displacement, and the tissue was embedded in paraffin wax for morphometric analysis. Sections were serially cut (5 μm) throughout the whole tissue and 10 sections every 100 sections were collected and mounted on polysine glass slides. The lung sections were stained with hematoxylin and eosin for the determination of alveolar structure, or Miller's elastin stain for the measurement of elastin content.

Stereological analysis of lung samples was performed blind to the treatment group using a light microscope (Olympus U-Spt) equipped with a computer-assisted stereology system (CAST 2.0; Olympus). Using point counting with a grid containing 8 × 8 points and 20 fields of view per section in 10 sections at ×40 magnification with a minimum of 200 counts, the percentages of section occupied by alveolar airspace (alveolar lumen), alveolar tissue (alveolar walls), extra-alveolar airspace (bronchial and bronchiolar airspaces), and extra-alveolar (bronchial, bronchiolar, vascular, and connective tissue). Alveolar wall thickness was measured using orthogonal intercepts, adjusted for tissue shrinkage, and was expressed as the harmonic mean value in 2 sections per animal using 6 lines per field of view and 20 fields of view per section at ×40 magnification. Alveolar surface area per unit volume (S_v_), also adjusted for tissue shrinkage, was measured using stereological cycloids (8 × 8 cycloids in 10 fields of view at ×40 magnification) and was calculated using the next equation: S_v_ = 2(ΣI)/(l/p(ΣP)), where ΣI is the sum of the intersections, ΣP is the sum of points on lung tissue, and l/p is the length of the cycloid per point (26 μm) (26). In addition, the volume fraction of elastin in interalveolar septae and secondary septal crests, and the density of secondary septal crests corrected for tissue shrinkage, were measured in 5 sections by point counting using a 10 × 10 grid and 20 fields of view per section at ×40 magnification.

### Biochemical and molecular analyses

#### Plasma hormone concentrations and pulmonary glycogen content

Plasma leptin concentration was measured by RIA using ovine leptin standards as described previously (10, 27); the lower limit of detection was 0.09 ng/mL and the interassay coefficient of variation was 5%. Total plasma cortisol concentration was measured by ELISA (IBL International); the sensitivity of the assay was 2.5 ng/mL and the interassay coefficient of variation was less than 8%. Pulmonary glycogen content was determined using an enzymatic method as described previously (28) and was expressed as milligrams of glucose per gram of lung tissue.

#### Quantitative RT-PCR

Tissue RNA was isolated from 15-mg samples of fetal lung (saline n = 6; 0.5 LEP n = 10; 1.0 LEP n = 7), and reverse transcription was carried out as described previously (29). TaqMan quantitative real-time-PCR was performed to measure mRNA abundance of target genes. Samples were analyzed using a TaqMan 7900HT, and data were acquired and processed with Sequence Detector v.2.3 software (Applied Biosystems) (29). The ovine mRNA probes and primers for leptin, the active long form of the leptin receptor (Ob-Rb), and all forms of the leptin receptor (Ob-R), elastin, VEGF-A and VEGFR2, ACE, PPARγ, bovine PTHrP, and SP-A, SP-B, and SP-C, and bovine SP-D are shown in Table 1. Each sample was measured in triplicate and normalized to the geometric mean of two housekeeping genes, glyceraldehyde-3-phosphate dehydrogenase and cyclophilin A (Table 1). The mRNA levels of these housekeeping genes were not affected by leptin treatment. For each plate, a negative control without cDNA was used to ensure that no amplicon contamination had occurred in the reaction, and a single cDNA sample was measured on each plate across all assays as a quality control. In order to compare mRNA abundance of target genes, cycle thresholds (Ct) determined by qRT-PCR were analyzed using the δδCt method as all standard curves were linear and parallel.

**Table 1.** Primer and Reporter Sequences Used for TaqMan qRT-PCR in the Sheep

Gene	Forward Primer Sequence	Reverse Primer Sequence	Reporter Sequence	Reporter Dye
Leptin	CAAGACGATTGTCACCAGGATCAA	CCAGTGACCCTCTGTTTGGA	TCACACACGCAGTCCGT	FAM
Ob-Rb (long form)	GGAGACAGCCCTCTGTTAAATATGC	TGAGCTGTTTATAAGCCCTTGCT	CCTCCTCGGCTTCACC	FAM
Ob-R (all isoforms)	GAGCGCCCTTCTTACCTTTACTA	CCAACCGCTGTCAGAATTTTAGGT	CACAAGATGTCATATATTTTC	FAM
SP-A	GAGCCTGGCGAGAGAGG	CTTGATGTCTGATCTCATGGAGTGT	TCCTCCAGGGTTTCCAG	FAM
SP-B	AGCCTGGGCCTCAGACA	CGGCAGAGCCAGCAGAA	CAGCAGCGGTTCCCCA	FAM
SP-C	GCAGCAAAGAGGTCTTGATGGA	GATGAGAAGGCGTTTGATGTTCAC	CCGCCGGACTACTC	FAM
SP-D (bovine)	TaqMan(R) gene expression assays (assay ID, Bt03217554_mH; part number, 4 331 182)			FAM
Elastin	GCTGCAGCTGCTAAAGCA	GGCTCCTAGGACACCTCCAA	CCAGCGGCACCAAAT	FAM
VEGF-A	GCTCTCTTGGGTGCATTGGA	GCCTGGGACCACTTGGC	CCTTGCCTTGCTGCTCTACCTTCACCA	FAM
VEGF-R2	GAACTAGAATGAGAGCCCCTGATT	CCCCATGCCAGCAGTCAA	ATGGTCTGGTACATTTC	FAM
ACE	CCTTCCCGCTACAACTATGACT	GGACAACCGGAGGACAGATC	ATACTTGGTTCGAAGATACC	FAM
PPARγ	CGATTCCAGAAGTGCCTTGCT	GAGATCTCTGCTAACAGCTTTTCCT	CCAAACCTGATGGCATTAT	FAM
PTHrP (bovine)	TaqMan(R) gene expression assays (assay ID, Bt03224326_m1; part number, 4 351 372)			FAM
GAPDH	GCTACACTGAGGACCAGGTT	AGCATCGAAGGTAGAAGAGTGAGT	CTCCTGCGACTTCAAC	FAM
Cyclophilin A	GGTTCCTGCTTTCACAGAATAATTCC	GTACCATTATGGCGTGTGAAGTCA	CACCCTGGCACATAAA	FAM

Abbreviation: GAPDH, glyceraldehyde-3-phosphate dehydrogenase.

#### Western blotting

The pulmonary protein contents of Ob-Rb and the total and phosphorylated forms of one of its signaling molecules, the signal transducers and activators of transcription-3 (STAT3), were determined by Western blotting. Frozen samples of fetal lung (100 mg) were homogenized, and the protein concentration of the lysates were measured as described previously (29). Equal amounts of sample protein were separated using 7.5% Mini-PROTEAN precast gels followed by incubation overnight at 4°C with rabbit polyclonal primary antibodies (Table 2) to 1) the ovine long-form leptin receptor (Ob-Rb) (2-μg/mL orb6312; Biorbyt); 2) the mouse phosphorylated STAT3 containing Ser727 (0.5-μg/mL sc-8001-R; Insight Biotechnology Ltd); or 3) the human STAT3 (1:500, 07-2173; Millipore) (29). Protein expression was visualized using ECL Plus chemiluminescence system according to the manufacturer's instructions (Amersham Biosciences) and quantified using ImageJ software (National Institutes of Health; http://rsb.info.nih.gov/ij/) after normalization to Ponceau S staining (30).

**Table 2.** Antibody Table

Peptide/Protein Target	Antigen Sequence (if Known)	Name of Antibody	Manufacturer, Catalog Number, and/or Name of Individual Providing the Antibody	Species Raised in; Monoclonal or Polyclonal	Dilution Used
Ovine long-form leptin receptor	KLH-conjugated synthetic peptide derived from sheep leptin receptor C terminus	Antileptin receptor	Biorbyt, orb6312	Rabbit; polyclonal	2 μg/mL
Mouse phosphorylated STAT3 containing Ser727		Antiphosphorylated STAT3 (Ser727)	Santa Cruz Biotechnology, Inc, sc-8001-R	Rabbit; polyclonal	0.5 μg/mL
Human STAT3		Anti-STAT3	Millipore, 07-2173	Rabbit; polyclonal	1:500

### Statistical analysis

Data are presented as mean ± SEM. All data were analyzed for normality, and parametric and nonparametric tests were used as appropriate (SPSS). The effect of the sex of the fetus on all data was initially assessed using a two-way ANOVA with sex and treatment as factors; no significant sex differences were observed and therefore, values from male and female fetuses were analyzed together in each treatment group. Data obtained from the 3 groups were compared by one-way ANOVA followed by Tukey's post hoc test or Kruskal-Wallis ANOVA on Ranks followed by Dunn's test. Gene expression data were expressed as fold changes in 2^−(δδCt)^ values relative to the mean value of the saline group. Repeated measures ANOVA (9 levels) was used to analyze the deflation limb of the lung curves for each treatment compared with the saline group. Repeated measures ANOVA (3 levels) was used to analyze the plasma concentrations of leptin and cortisol for each treatment compared with the saline group. Within the saline-treated group, relationships between plasma leptin concentration on day 5 of infusion and indices of pulmonary structure and function were assessed by linear regression. Differences where *P* < .05 were regarded as significant.

## Results

### Plasma hormone concentrations and blood gas analyses

**Figure 1.**
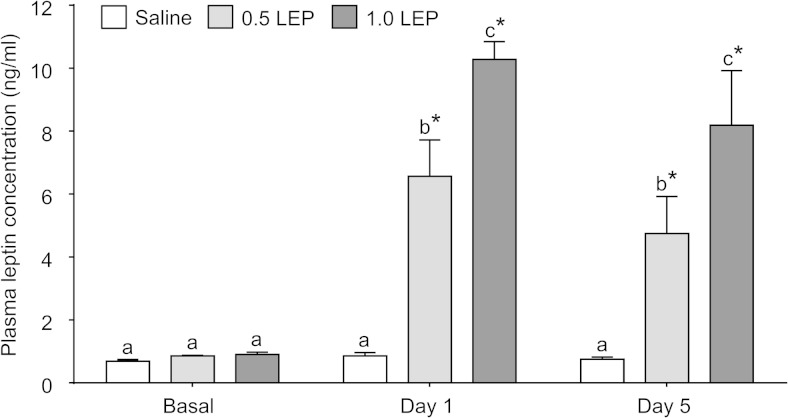
Mean ± SEM plasma leptin and cortisol concentrations in sheep fetuses infused iv with either saline or leptin (0.5 or 1.0 LEP) for 5 days. Basal concentrations represent the mean value from the 3 days before the start of infusion. Day 1 is the first day after infusion starts and day 5 is the last day of infusion before delivery and tissue collection. On each day, values with different letters are significantly different from each other, *P* < .05. *, significant difference from basal values, *P* < .05.

There was no difference in basal plasma leptin concentration between the treatment groups before the infusion period (Figure 1). On the first day after the commencement of the infusion, circulating leptin concentrations increased significantly in the fetuses treated with 0.5 LEP and 1.0 LEP, above respective basal concentrations and to concentrations higher than those seen in the saline group (*P* < .05 in all cases) (Figure 1). In the 0.5 LEP and 1.0 LEP groups, plasma leptin remained elevated throughout the 5-day period of infusion (*P* < .05 on all days) (Figure 1). There was a significant interaction between treatment and time on plasma leptin concentration (*P* < .001), and plasma leptin in the 1.0 LEP group was significantly higher than in the 0.5 LEP group throughout the infusion period (*P* < .05 on all days) (Figure 1). Plasma leptin concentration remained unchanged in the saline-infused fetuses over all days of study (Figure 1).

Over the period of infusion, no significant differences in plasma cortisol were observed either within or between each treatment group, and no significant interactions between treatment and time were observed (Table 3). Arterial blood pH, partial pressure of oxygen, partial pressure of carbon dioxide, O_2_ saturation, hemoglobin content, and glucose and lactate concentrations, in the ewe and fetus were not affected by leptin infusion and did not differ between treatment groups on any day of the study (data not shown).

**Table 3.** Mean ± SEM Values for Animal Morphometry, Fetal Plasma Cortisol, and Lung Measurements in the 3 Treatment Groups

	Saline (n = 13)	0.5 LEP (n = 10)	1.0 LEP (n = 7)
Maternal body weight (kg)	44.8 ± 2.2	44.1 ± 2.4	45.6 ± 3.8
Total placentome weight (g)	319 ± 22	298 ± 18	267 ± 35
Fetal body weight (kg)	2.76 ± 0.16	2.74 ± 0.12	2.32 ± 0.19
CRL (cm)	43.0 ± 1.0	43.5 ± 0.7	41.4 ± 1.1
BMI (kg/m^2^)	14.8 ± 0.4	14.4 ± 0.5	13.3 ± 0.4
PI (kg/m^3^)	34.4 ± 0.9	33.2 ± 1.2	32.2 ± 0.5
Plasma cortisol concentration (ng/mL)			
Basal	8.4 ± 1.2	6.6 ± 0.8	6.1 ± 1.4
Day 5	10.4 ± 2.1	7.1 ± 1.3	12.0 ± 3.7
Absolute lung weight (g)	74.4 ± 4.5	74.1 ± 5.2	64.0 ± 4.3
Relative lung weight (% of body weight)	2.71 ± 0.10	2.71 ± 0.16	2.80 ± 0.13
Pulmonary water content (%)	89.1 ± 0.2	89.1 ± 0.4	89.3 ± 0.4
Pulmonary glycogen content (mg/g)	2.50 ± 0.28 (n = 6)	2.15 ± 0.16	2.54 ± 0.37
Alveolar air-space (%)	49.7 ± 2.8	54.8 ± 0.7	53.0 ± 3.5
Alveolar tissue (%)	44.4 ± 2.8	39.6 ± 0.8	38.7 ± 2.4
Extra-alveolar air-space (%)	2.7 ± 0.3	2.4 ± 0.2	3.0 ± 0.4
Extra-alveolar tissue (%)	3.2 ± 0.3	3.2 ± 0.3	5.4 ± 1.1
Alveolar wall thickness (harmonic mean, μm)	9.28 ± 0.66^a^	7.15 ± 0.25^b^	9.88 ± 0.43^a^
Alveolar surface area (cm^2^/cm^3^)	707 ± 139	535 ± 143	911 ± 388
Secondary septal crest density (number/mm^2^)	404 ± 33	548 ± 65*	527 ± 69
Relative volume of elastin			
Interalveolar septae	28.9 ± 1.5^a^	35.1 ± 1.8^b^	31.4 ± 2.0^a,b^
Secondary septal crests	11.2 ± 1.1	12.7 ± 1.4	11.6 ± 1.1
Total	40.2 ± 1.8^a^	47.8 ± 2.6^b^	43.0 ± 2.4^a,b^

For each variable, values with different letters (a, b) are significantly different from each other, one-way ANOVA, *P* < .05.

* Significantly different from saline group, unpaired Student's *t* test, *P* < .05.

### Body composition

No significant differences in the measures of fetal body composition (body weight, CRL, BMI, and PI) were observed after 5 days of 0.5 LEP or 1.0 LEP infusion compared with the saline group (Table 3). In addition, maternal body weight, absolute and relative fetal lung weights, and pulmonary glycogen and percentage water content, did not differ between the treatment groups (Table 3). Leptin infusion had no effect on the total weight of placentomes (Table 3), total uteroplacental weight (uterus, placentomes, and placental membranes) or the number, mean weight or type of placentomes (data not shown).

### Fetal lung function

**Figure 2.**
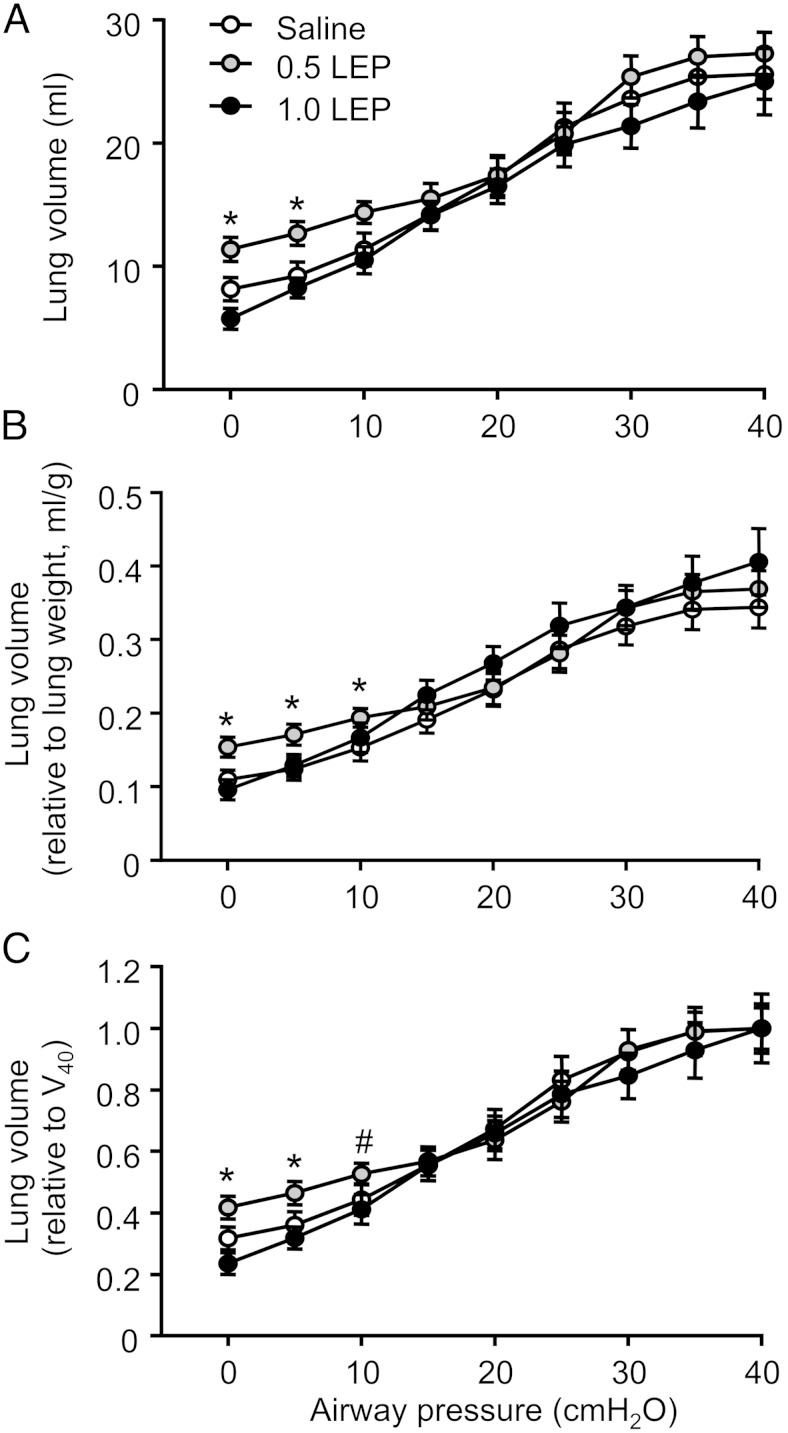
Mean ± SEM lung volumes with deflating airway pressure, expressed as (A) absolute values, (B) relative to lung weight, and (C) relative to V_40_ in sheep fetuses infused iv with either saline or leptin (0.5 or 1.0 LEP) for 5 days. *, significant difference from saline and 1.0 LEP group, *P* < .05 (#, *P* = .06).

Significant interactions between treatment and deflation pressure were observed for lung volume when it was expressed in absolute terms or relative to lung weight or V_40_ (*P* < .05 in all cases) (Figure 2). In the fetuses infused with 0.5 LEP, the absolute lung volumes at closing pressure (0 cmH_2_O) and at 5 cmH_2_O of the deflation limb of the pressure-volume curve were significantly greater than those seen in the fetuses infused with saline or 1.0 LEP (*P* < .05 in all cases) (Figure 2A). When corrected for lung weight, the relative lung volumes at 0, 5, and 10 cmH_2_O were significantly increased in the 0.5 LEP group compared with the saline or 1.0 LEP groups (*P* < .05 in all cases) (Figure 2B). The lung volume expressed relative to V_40_ was significantly greater in the 0.5 LEP fetuses compared with the saline or 1.0 LEP-infused fetuses at 0 and 5 cmH_2_O (*P* < .05 in both cases), with a tendency to be increased at 10 cmH_2_O (*P* = .06) (Figure 2C). The lung pressure-volume curve, using absolute or relative data, was not different between the 1.0 LEP and saline groups (Figure 2). There were no significant effects of either dose of leptin on V_40_ (Figure 2A) or on the slope of the deflation curves between 10 and 30 cmH_2_O (data not shown).

### Stereological analysis of the fetal lung

Alveolar wall thickness was significantly reduced in the fetuses infused with 0.5 LEP compared with those treated with saline and 1.0 LEP (*P* < .05) (Table 3). Secondary septal crest density was not affected by leptin administration when the data were analyzed by one-way ANOVA; however, when compared by unpaired Student's *t* test, the density of secondary septal crests in the lungs of fetuses treated with 0.5 LEP was significantly higher than that seen in the saline group (*P* < .05) (Table 3). The surface area density, the percentage volume fractions of alveolar airspace, alveolar tissue, extra-alveolar airspace and extra-alveolar tissue, were not affected by leptin infusion (Table 3). On day 5 of treatment, plasma leptin concentration in the saline-infused fetuses correlated positively with the percentage volume fraction of alveolar air-space (*r* = 0.74, n = 12, *P* < .05) and inversely with the percentage volume fraction of alveolar tissue (*r* = −0.71, n = 12, *P* < .05). The percentage volume fraction of elastin in the interalveolar septae, and hence the total volume fraction of elastin (elastin in the interalveolar septae and secondary septal crests), were significantly increased in the fetuses infused with 0.5 LEP compared with those infused with saline (*P* < .05 in both cases) (Table 3); the values in the fetuses infused with 1.0 LEP were intermediate to those treated with saline or 0.5 LEP (Table 3). There were no significant differences in volume fraction of elastin in the secondary septal crests between the treatment groups (Table 3).

### Pulmonary gene and protein expression

**Figure 3.**
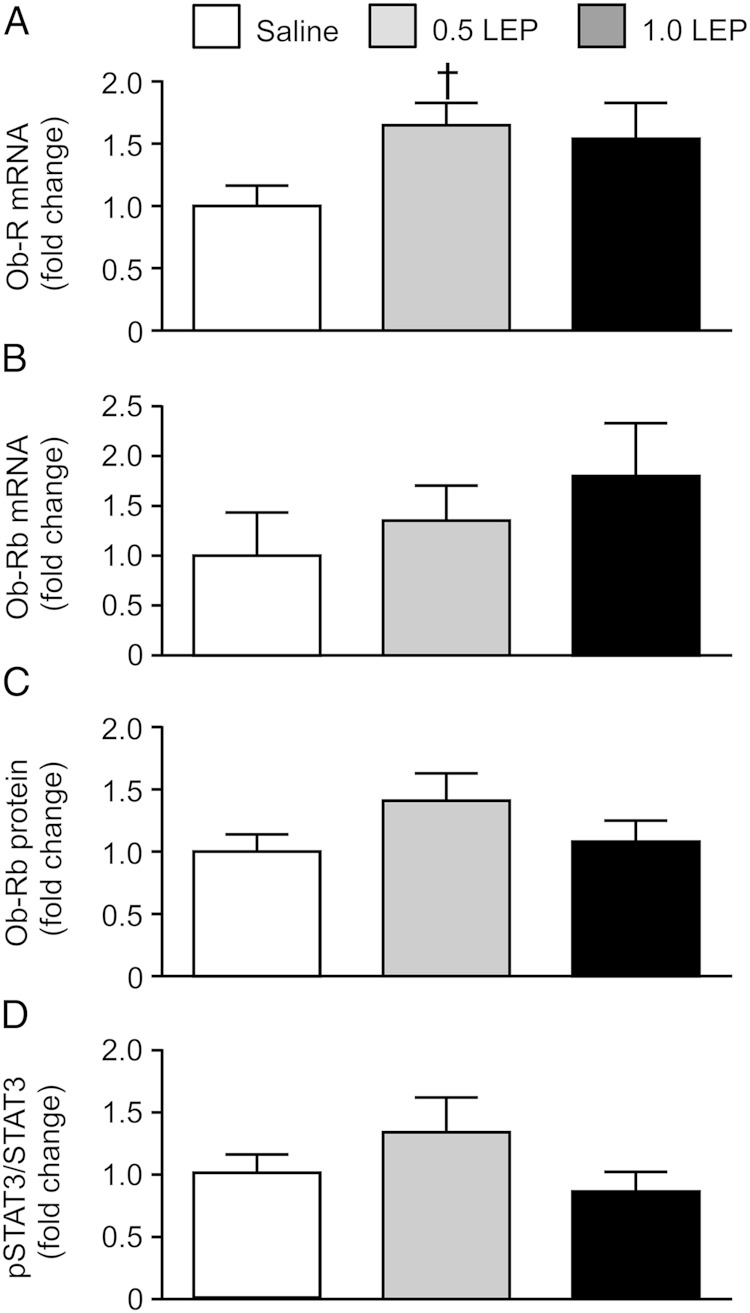
Mean ± SEM relative mRNA abundance of (A) all forms of the leptin receptor Ob-R and (B) the long-form leptin receptor Ob-Rb, and relative protein expression of (C) the long-form leptin receptor and (D) phosphorylated STAT3 relative to total STAT3, in the lungs of sheep fetuses infused iv with either saline or leptin (0.5 or 1.0 LEP) for 5 days. †, significantly different from saline group, unpaired Student's *t* test, *P* < .05.

**Figure 4.**
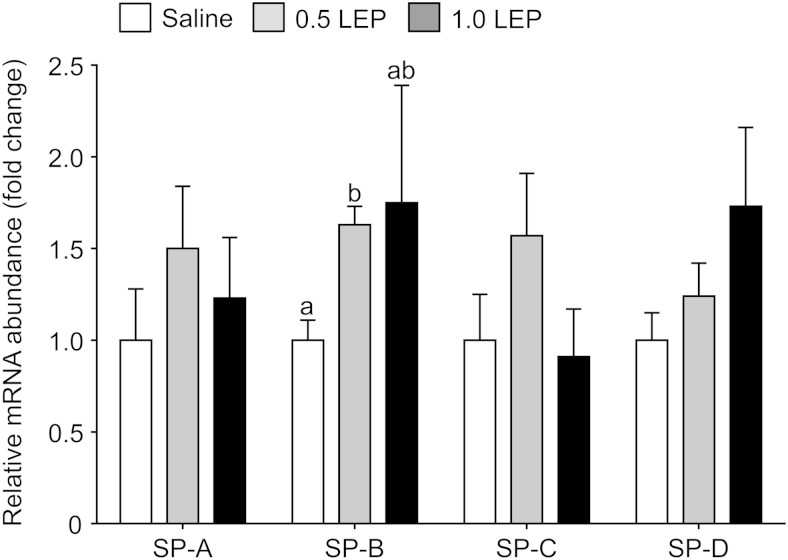
Mean ± SEM relative mRNA abundance of SP-A, B, C, and D in the lungs of sheep fetuses infused iv with either saline or leptin (0.5 or 1.0 LEP) for 5 days. For each gene, values with different letters are significantly different from each other, one-way ANOVA, *P* < .05.

Using primers that recognized all forms of the leptin receptor, Ob-R mRNA abundance tended to increase in response to leptin treatment (*P* = .09, one-way ANOVA); when analyzed subsequently by unpaired Student's *t* test, pulmonary Ob-R mRNA level in the fetuses treated with 0.5 LEP was significantly greater than that seen in the saline group (*P* < .05) (Figure 3A). Transcript and protein levels of the long-form leptin receptor, Ob-Rb, and the phosphorylated STAT3 to STAT3 protein ratio, in the fetal lungs were not affected by 0.5 LEP or 1.0 LEP treatment (Figure 3, B–D). Pulmonary mRNA abundance of SP-B was significantly elevated in the fetuses of the 0.5 LEP group compared with those in the saline group (*P* < .05) (Figure 4); however, SP-B mRNA level in the 1.0 LEP fetuses remained intermediate to the level measured in the saline and 0.5 LEP fetuses (Figure 4). The mRNA levels of SP-A, SP-C, SP-D, elastin, VEGF-A, VEGFR2, ACE, PTHrP, and PPARγ in the fetal lungs were not affected by leptin administration (Figure 4 and Table 4). Leptin mRNA was not detected in the lungs of the sheep fetuses treated with saline or leptin. In the saline-infused fetuses, plasma leptin concentration on day 5 was positively correlated with SP-D mRNA (*r* = 0.93, n = 5, *P* < .05) and inversely correlated with PTHrP mRNA levels (*r* = −0.98, n = 5, *P* < .005).

**Table 4.** Mean ± SEM Relative mRNA Abundance in the Lungs of Sheep Fetuses Infused iv With Either Saline or Leptin (0.5 or 1.0 LEP) for 5 Days

	Saline (n = 6)	0.5 LEP (n = 10)	1.0 LEP (n = 7)
Elastin	1.00 ± 0.24	0.85 ± 0.16	1.13 ± 0.50
VEGF-A	1.00 ± 0.11	0.81 ± 0.14	0.64 ± 0.12
VEGFR2	1.00 ± 0.23	0.56 ± 0.12	0.50 ± 0.12
ACE	1.00 ± 0.19	0.76 ± 0.16	1.02 ± 0.30
PPARγ	1.00 ± 0.17	0.83 ± 0.08	1.11 ± 0.28
PTHrP	1.00 ± 0.16	1.28 ± 0.27	0.91 ± 0.15

Data are expressed as a fold change relative to the saline group.

## Discussion

This study has demonstrated for the first time, in a species like the human where alveolar development occurs before birth, that leptin administration matures aspects of lung structure and increases pulmonary expression of the SP-B gene involved in surfactant production. Specifically, 0.5 LEP treatment in fetal sheep, that elevated circulating leptin concentration by 5- to 7-fold for 5 days, caused a reduction in alveolar diffusion distance and increased the pulmonary volume at closing pressure, the density of secondary septal crests, elastin content in alveolar walls, and the transcript levels of Ob-R and SP-B. Importantly, these effects of leptin on lung function and maturation occurred without any change in plasma cortisol concentration, which is well known to promote pulmonary maturation before birth (3). Overall, the findings of the present study support a key role for leptin in developmental and glucocorticoid-induced lung maturation in the fetus near term.

Leptin exposure in utero had moderate, but potentially functional, consequences for the deflation limb of the pressure-volume curve measured in the ovine fetal lungs. No change was observed in V_40_ or static lung compliance, but the elevated plasma leptin concentration in the 0.5 LEP group was associated with an increase in the lung volume at 0 cmH_2_O closing pressure. This finding suggests that the fetal lungs remained in a more expanded state at the end of deflation, which may be a function of the elastic properties of the lung tissue and/or the surface tension forces and surfactant production by type II pneumocytes (31). Indeed, in the 0.5 LEP group, the increase in lung volume at closing pressure was associated with a significant increase in pulmonary elastin content in the alveolar walls, although no change in elastin mRNA abundance was observed in response to leptin treatment. In addition, thinning of the alveolar walls was induced by 0.5 LEP treatment. Therefore, leptin may contribute to the control of structural remodeling of AT, and the development of the elastic properties, that occurs in the fetal lung during late gestation.

Previously, Ob-Rb protein has been localized to type II pneumocytes in fetal ovine lungs, especially close to term, which suggested that leptin may play a role in the activation of surfactant production (29). In the present study, the mRNA abundance of SP-B was up-regulated by 0.5 LEP treatment, although transcript levels of the other SPs were unaffected by leptin treatment. Surfactant phospholipid content was not determined in the present study and measurements of saturated phosphatidylcholine and SP protein expression would be necessary to confirm the synthesis and secretion of surfactant in response to leptin administration in utero. However, the present findings support, in part, those obtained previously from a series of studies on fetal tissue in vitro. In lung explants obtained from fetal rats, physiological concentrations of leptin increased mRNA abundance of SP-A, SP-B, and SP-C, and protein levels of SP-A and SP-B, and the synthesis of surfactant phospholipids (17, 19). In addition, antenatal treatment of pregnant rats with leptin increased type II pneumocyte number and SP expression in the fetal lungs (19, 32). In the lungs of *Xenopus laevis* tadpoles cultured in vitro, leptin exposure reduced the thickness of the alveolar walls and increased lamellar body size in association with dose-dependent increases in both secreted SP-B and C (33). In contrast, a study that administered leptin to fetal sheep by an ultrasound-guided single im injection did not show any changes in pulmonary function or SP synthesis (34); however, in that experiment, recombinant human leptin was used, and it was difficult to assess the effectiveness of treatment as plasma leptin concentration in the fetus was not determined. In addition, fetal delivery and tissue collection took place 3 days after the single injection of leptin, at a time when leptin may have been cleared from the circulation. In sheep, recombinant human leptin injected sc had an estimated circulating half-life of 4 hours (35). In the present study, the iv infusion of recombinant ovine leptin elevated leptin levels in the fetal circulation from the first to the fifth day of treatment.

In rodent species, leptin has been proposed as an important signaling molecule in the paracrine interactions between type II pneumocytes and lipofibroblasts that provide the lipid substrates for surfactant synthesis. Stretch of the type II pneumocyte induces the release of PTHrP, which acts on neighboring lipofibroblasts to stimulate uptake, storage and trafficking of triglycerides mediated by the actions of PPARγ and leptin (21, 22). Studies using fetal rat tissue in vitro have shown that dexamethasone and PTHrP increase leptin mRNA abundance in pulmonary lipofibroblasts and that PTHrP-induced phospholipid synthesis is inhibited by an antileptin antibody in a coculture of type II pneumocytes and lipofibroblasts (19). In the sheep fetus, however, the absence of leptin mRNA in the lungs at 130 days of gestation (29), and the lack of effect of leptin treatment on PTHrP and PPARγ genes in the present study, suggests that the maturational effects of leptin via Ob-Rb are likely to be endocrine, rather than by local synthesis and paracrine actions of the hormone as reported in the rodent species.

In the current study, the lower dose of recombinant ovine leptin (0.5 LEP) was more effective than the higher 1.0 LEP dose in promoting aspects of lung maturation in utero. Although significant responses were observed in the fetuses treated with 0.5 LEP, the higher dose of leptin had no effect on the lung volume at closing pressure, alveolar wall thickness, pulmonary elastin content or the mRNA level of SP-B. The reasons for differences between the two treatment groups are unclear, although in several cases, larger interanimal variation may have contributed to the lack of statistical significance in the values from the 1.0 LEP group. The circulating concentration of leptin achieved in the 1.0 LEP group was significantly greater than that seen in the 0.5 LEP group, and levels in both groups of fetuses were higher than those normally seen in fetal sheep near term (10). Exposure to supraphysiological leptin concentrations in utero may have influenced pulmonary Ob-R expression and postreceptor signaling to different extents in the two treatment groups. No significant differences in Ob-Rb or phosphorylated STAT3/STAT3 protein levels were observed between the fetuses infused with leptin or saline, although an increase in Ob-R mRNA abundance was induced by 0.5 LEP treatment; these findings suggest changes in the control of Ob-R gene transcription, protein translation and/or receptor protein degradation which require further investigation. Of the six known splice variants of the Ob-R gene, only the long form of the leptin receptor, Ob-Rb, contains intracellular motifs that can activate the Janus kinase/STAT intracellular signal transduction pathway (36). Phosphorylation of STAT3 results in the formation of an active dimer which translocates to the cell nucleus and binds to the promoter region of target genes to control gene expression (36). STAT3 is expressed in various cell types in the developing lung, and studies in vitro have demonstrated that STAT3 can activate the promoter on the SP-B gene (37). In addition, differences in responses between the two leptin groups may relate to changes in the pulmonary metabolism of cortisol and expression of the glucocorticoid receptor which also remain to be determined. Furthermore, the extent to which the actions of leptin on pulmonary development before birth are mediated by immune and/or central nervous pathways is unknown.

Intravenous infusion of recombinant ovine leptin in the sheep fetus for 5 days was capable of mimicking some of the effects of endogenous and exogenous glucocorticoids on lung development in utero. However, leptin treatment did not influence pulmonary mRNA abundance of ACE or SP-A, -C or -D, or the relative volumes of alveolar air-space and tissue, which are known to be affected by glucocorticoids before birth (3, 38, 39). The lack of effect of leptin on the relative proportions of placentome types also indicates that the effects of cortisol on placental morphology (25) occur independent of the actions of leptin. A greater understanding of the mechanisms by which glucocorticoids exert their maturational effects on the fetal lungs is crucial for the development of specific therapeutic agents for the treatment of the respiratory consequences of prematurity. This is of particular importance because there is growing evidence that antenatal synthetic glucocorticoids have wide-ranging effects in the offspring with potential long-term consequences for postnatal health and disease (40).

The findings of the present study also have implications for lung development in macrosomic offspring. Umbilical cord leptin concentration correlates with adiposity in human neonates (41, 42) and babies born to obese mothers, and to those with gestational or type 1 diabetes, have higher umbilical leptin concentrations at delivery compared with those born to lean, nondiabetic mothers (43–45). These infants are at increased risk of RDS in the neonatal period (46), an observation that appears to be inconsistent with the findings of the present study. Impaired lung development in the offspring of obese and/or diabetic mothers may be due primarily to the effects of high circulating concentrations of glucose and insulin in utero (47), which offset the potential maturational effects of leptin. It is also possible, however, that longer term exposure to leptin before birth may influence pulmonary Ob-Rb expression and leptin signaling with adverse consequences for lung maturation and respiratory function at birth. In adult life, chronic exposure to leptin associated with obesity leads to resistance to the appetite-regulatory actions of leptin at the hypothalamus (48, 49) and it may be speculated that leptin insensitivity could arise in fetuses overexposed to leptin. The present study has demonstrated beneficial effects of leptin treatment on aspects of lung development in the ovine fetus, at least over a 5-day period and with a 5- to 7-fold increase in plasma leptin concentration. The consequences of longer term leptin exposure in utero need further investigation.
